# Assessment of Optical Properties and Monte Carlo-Based Simulation of Light Propagation in Blackhearted Potatoes

**DOI:** 10.3390/s25123713

**Published:** 2025-06-13

**Authors:** Yalin Guo, Yakai He, Xilong Li, Zhiming Guo, Mengyao Zhang, Xiaomei Huang, Zhiyou Zhu, Huabin Jian, Zhilong Du, Huangzhen Lv

**Affiliations:** 1 Chinese Academy of Agricultural Mechanization Sciences Group Co., Ltd., Beijing 100083, China; mumu1119@outlook.com (Y.G.); lxl879684795@163.com (X.L.); zmytumy@gmail.com (M.Z.); xlyjianhuabin@163.com (H.J.); 2 China National Packaging and Food Machinery Corporation, Beijing 100083, China; hlyakai@163.com (Y.H.); 13463669512@163.com (Z.D.); 3 School of Food and Biological Engineering, Jiangsu University, Zhenjiang 212013, China; guozhiming@ujs.edu.cn; 4 Key Laboratory of Agro-Products Primary Processing, Ministry of Agriculture and Rural Affairs of China, Beijing 100083, China; huangxiaomei857@163.com (X.H.); zzy66608@163.com (Z.Z.)

**Keywords:** blackhearted potatoes, Vis-NIR spectroscopy, optical properties, light propagation, Monte Carlo simulation

## Abstract

This study investigated the optical properties (OPs) and Monte Carlo (MC) simulations of light propagation in Healthy Group (HG) and Blackhearted Group (BG) potatoes. The MC simulation of light propagation indicated that both the photon packet weight and the penetration depth were significantly lower in blackhearted tissues than in healthy tissues. The simulation revealed deeper light penetration in healthy tissues than in the blackhearted tissues, approximately 6.73 mm at 805 nm, whereas the penetration depth in blackhearted tissues was much shallower (1.30 mm at 805 nm). Additionally, the simulated absorption energy at both 490 nm and 805 nm was higher in blackhearted tissues, suggesting that these wavelengths effectively detect blackheart in potatoes. The absorption (*μ*_a_) and reduced scattering (*μ*’_s_) coefficients were obtained using Vis-NIR spectroscopy, which represented a notable increase in *μ*_a_ in BH tissues, particularly around 550–850 nm, and an increase in *μ*’_s_ across the Vis-NIR region. Based on transmittance (*T*t), *μ*_a_ and *μ*’_s_, Support Vector Machine Discriminant Analysis (SVM-DA) models demonstrated exceptional performance, achieving 95.83–100.00% accuracy in Cross-Validation sets, thereby confirming the robustness and reliability of the optical features for accurate blackheart detection. These findings provide valuable theoretical insights into the accuracy and robustness of predictive models for detecting blackhearted potatoes.

## 1. Introduction

Potato (*Solanum tuberosum* L.) is a staple crop that is important for global food security. As a leading potato producer, China faces significant challenges due to compromised yield and quality [1]. Among various postharvest issues, blackheart (BH) is a physiological disorder affecting potato tubers in which internal tissue discolors during storage [2]. Bartholomew initially identified BH in shipped potatoes [3] and it continues to result in significant losses for the potato industry [4]. BH primarily affected affects medullary tuber tissues [2,5]. Affected tubers remain firm and odorless, without external symptoms. The disorder becomes detectable only when tubers are cut open or show severe internal decay. Thus, tubers may pass quality control checks and be marketed with defects.

Researchers have increasingly employed rapid, non-destructive techniques to monitor quality attributes of agricultural products such visible and near-infrared (Vis-NIR) spectroscopy [6,7,8], Hyperspectral imaging [9], and Machine Vision [10]. Vis-NIR spectroscopy has shown a strong potential for detecting internal disorders such as blackheart in agricultural products [11,12]. Different wavelengths exhibit distinct penetration capabilities in biological tissues. Compared with short-wave infrared (SWIR) and ultraviolet (UV) light, near-infrared (NIR) light demonstrates a greater penetration depth, thereby facilitating the acquisition of internal structural information [9]. However, these detection methods cannot be widely applied due to inspection accuracy and stability limitations. Light transport in biological tissues and its interaction with tissues is the basis for spectral, non-destructive testing [12]. Quantifying the optical properties (OPs), such as light propagation paths and distribution, penetration depth, absorption coefficient (*μ*_a_), and reduced scattering coefficient (*μ*’_s_) between healthy and blackhearted potato tissues, is essential for enhancing internal quality detection. The *μ*_a_ reflects the chemical composition, including soluble sugars, acids, pigments, and water content, whereas the *μ*’_s_ represents structural properties such as porosity, pore distribution, and sizes [11,13]. Furthermore, light propagation in biological tissues mainly involves photon interactions with the scattering and absorbing particles, which result in a limited penetration depth. Monte Carlo (MC) simulations are widely recognized as the gold standard for modeling light transport in turbid media, particularly biological tissues, due to their ability to accurately represent stochastic photon interactions governed by tissue optical properties. Previous research has predominantly concentrated on the optical properties and simulation of light transport in tissues infected by *Penicillium italicum* [14,15]. There is a significant lack of research concerning light propagation in internal-quality-deteriorated potatoes. Similarly, the OPs in cross-variety potatoes have also been scarcely studied. Current studies offer limited insight into agricultural products’ precise light transmission pathways, particularly their spatial distribution and attenuation across distinct tissue regions. This shortcoming restricts the practical application of spectroscopic methods in the non-destructive evaluation of internal quality in agricultural products [16].

In this study, we performed OPs measurements and chemical analyses on four potato cultivars and employed MC simulations to further characterize internal tissue behavior to model light transport in blackhearted regions. The objectives of this work were to (i) compare the structural characteristics of healthy and blackhearted tissues, (ii) assess OP variations during deterioration, and (iii) simulate light propagation in blackhearted samples based on MC modeling.

## 2. Materials and Methods

### 2.1. Potato Samples

Favorita, Xisen No. 6, Atlantic, and V7 are widely cultivated potato cultivars with round or oval shapes and varying skin colors depending on the variety [17]. All samples were selected at similar maturity, with an average mass of 211.86 ± 38.36 g and an equatorial diameter of 58.11 ± 2.79 mm, and were free of infections and surface defects. After washing with sterile water to remove soil, 50 tubers per cultivar were dried, surface sterilized, and sealed in plastic bags. The samples were then incubated at 38.5 °C for 48 h to induce internal discoloration, followed by refrigeration at 4 °C for 48 h. Subsequently, they were held at room temperature for 24 h before analysis. Twenty tubers per cultivar were selected to form the Healthy and Blackhearted Groups, while 15 additional tubers formed the Slightly Blackhearted Group. All selected tubers were used for OPs and physical and chemical analyses.

### 2.2. Optical Properties Measurements

**Figure 1:**
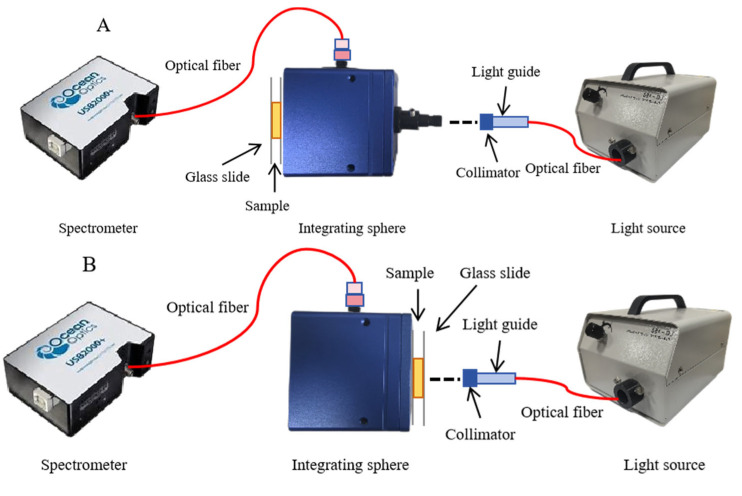
Schematic diagram of the single-integrating sphere system. (**A**) total diffuse reflectance mode; (**B**) total transmittance mode.

The single-integrating sphere (SIS) system (Figure 1) with the inverse adding doubling (IAD) algorithm was used to obtain the OPs. Figure 1A,B illustrates total diffuse reflectance and total transmittance, respectively. The SIS setup mainly comprised an integrating sphere (RK-36T, Ruike photoelectric, Guangzhou, China) with a diameter of 36 mm and a 9 mm sample port diameter with an inner surface coated with polytetrafluoroethylene (PTFE), which has an average reflectivity of 0.90, a 150 W adjustable power halogen lamp (LG-150, Wuling Optics, Shanghai, China), and a Vis-NIR spectrometer with a range of wavelengths from 400 to 1000 nm (USB2000+, OceanOptics, Orlando, FL, USA).

**Figure 2:**
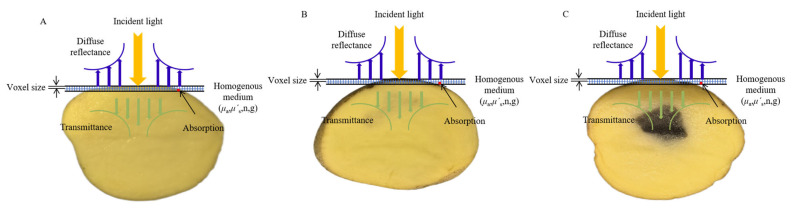
Light interaction with potato tissues. (**A**) Healthy Group. (**B**) Slightly Blackhearted Group. (**C**) Blackhearted Group.

All the measurements were conducted within a light-proof enclosure to avoid interference from ambient light. According to the results of pre-experimentations, approximately 2 mm thick inner medulla tissues from potatoes were finally selected as the sample slice (Figure 2). Each slice was used to acquire the reflectance and transmittance signals by adjusting the reflection and transmission modes of the DIS. The reflectance (*R*t) and transmittance (*T*t) fractions of potato slices were calculated and used to calculate the OPs with the IAD algorithm, which was described in detail in Scott Prahl [18].

### 2.3. Physicochemical Indicators

All physicochemical indicators were conducted on the same sampling region previously used for OPs. The color of each potato was measured at a fixed position using a digital colorimeter (CR-10 Plus, Konica Minolta Sensing, Inc., Tokyo, Japan). The color was expressed in the CIE L*a*b color space, where L* is an indicator of the lightness from black (0) to white (100), a* represents the tendency from green (negative value) to red (positive value), and b* represents the tendency from blue (negative value) to yellow (positive value) [19,20]. ΔL represents the difference in lightness, while Δa and Δb denote the chromatic differences along the red–green and yellow–blue axes, respectively. The overall color difference, ΔE, is calculated as the Euclidean distance in the CIE Lab* color space.

Dry matter (DM) content was determined by placing approximately 2 g of potato tissue into a pre-weighed aluminum dish. Samples were dried in an oven at 105 °C, weighed, and re-dried until the mass difference between consecutive measurements was less than 2 mg, indicating a constant weight.

Scanning electron microscopy (SEM) (Zeiss Sigma 300, Oberkochen, Germany) was conducted to analyze the microstructure of potato tissues from the Healthy and Blackhearted Groups. Each sample was sectioned into slices measuring 10 mm × 10 mm × 2 mm (width × height × depth) using a razor blade and subsequently dried at 80 °C to constant weight. One representative slice was dehydrated, freeze-dried, and further trimmed, then sputter-coated with gold prior to SEM imaging, following the protocol outlined by Wang et al. [21].

### 2.4. Penetration Depth of Potato Tissues

Based on the derived Ops, the light penetration depth (*σ*) can be calculated using the mean values of the *μ*_a_ and *μ*’_s_ at each wavelength. The penetration depth depended on light attenuation in biological tissues, which involved absorption and scattering [22]. This served as a useful indicator of light propagation in potato tissues. Taking 1 as the incident irradiance, the penetration depth is the level at which the incident light is reduced to 1/e (~37%) in the tissue [23]:

(1) $$\sigma =\frac{1}{\sqrt{3\mu_{a}(\mu_{a}+{\mu^{\prime }}_{s})}}$$

### 2.5. Monte Carlo Simulation in Homogeneous Tissues

**Figure 3:**
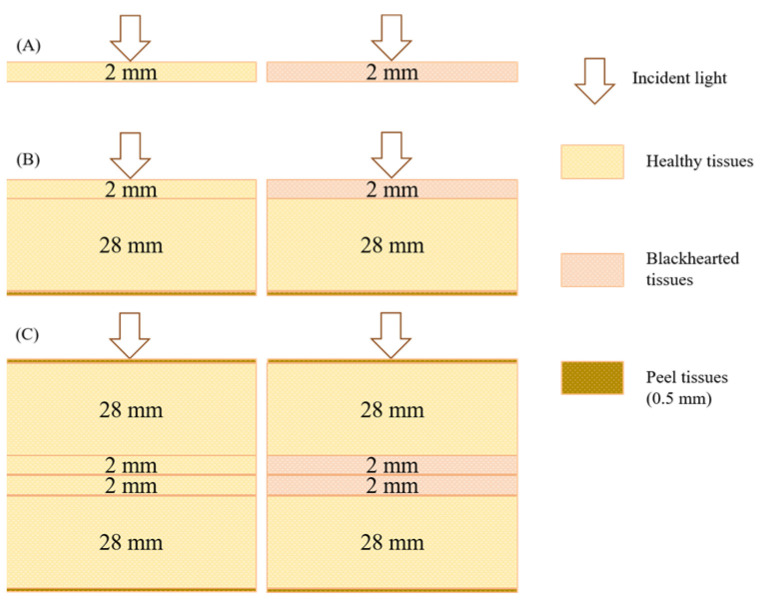
Simulation models of potato tissue. ((**A**): A 2 mm thick slice model; (**B**): A half-potato model; (**C**): A full-potato model).

As illustrated in Figure 3, three simulation models were developed to represent different tissue configurations. (A) A 2 mm thick slice model simulated photon distribution within isolated healthy and blackhearted tissue slices. (B) A half-potato model was constructed by placing a 2 mm layer of either healthy or blackhearted tissue atop a 28 mm healthy tissue block and 0.5 mm of peel tissue in order to examine light penetration and attenuation through superficial and subsurface layers. (C) A full-potato model was established by embedding two 2 mm slices of central tissue layer (healthy or blackhearted) between two 28 mm healthy tissue blocks externally wrapped with 0.5 mm peel tissues on both sides, simulating volumetric light propagation through the entire potato tissue structure.

Since photon–tissue interactions are primarily confined to a limited region, potato tissue was modeled as a semi-infinite homogeneous medium in the MC simulation framework [22]. Based on the average values of the estimated optical properties, Monte Carlo eXtreme (MCX), an open-source, voxel-based Monte Carlo simulation platform, was employed to simulate photon transport and compute optical attenuation characteristics [23]. The total energy distribution of photons in the 400–1000 nm spectral range was calculated to quantify the proportions of diffuse reflectance, transmittance, and absorption. Subsequently, two representative wavelengths corresponding to the minimal and maximal transmittance depth differences between healthy and blackhearted tissues were selected for further analysis. The absorption energy distribution and photon transmission trajectories within the tissue were simulated at these wavelengths to characterize potato tubers’ internal light transport behavior. In the MCX simulation process, the number of photons and the resolution were set to 100,000,000 and 70 × 70 × 70 mm, respectively; each detailed parameter in the MCX simulation process is shown in Table 1.

### 2.6. Statistic Processing and Analysis

Partial Least Squares Discriminant Analysis (PLS-DA) is a classical linear classification method that establishes a regression relationship between spectral variables and categorical class labels. In contrast, Support Vector Machine Discriminant Analysis (SVM-DA) is a nonlinear supervised learning algorithm that constructs optimal hyperplanes for sample separation in high-dimensional spaces. This study implemented PLS-DA and SVM-DA as supervised classification models to evaluate their effectiveness in distinguishing between healthy and diseased potato tissues. Model development and validation were conducted in PyCharm (2024, JetBrains, s.r.o., Prague, Czech Republic), with overall classification accuracy as the primary performance metric. The results were visualized using Origin 2025 (OriginLab Corporation, Northampton, MA, USA) and Matplotlib 3.8.2 in Python 3.9.5.

## 3. Results

### 3.1. The Changes in Quality Parameters

A significant increase in both ΔE and Δa values was observed in the Blackhearted Group across all four potato varieties, whereas a notable decline in ΔL and Δb values was detected within the same group. Specifically, the ΔE values of potatoes consistently rose as the healthy condition transitioned from the Healthy Group to the disease group, increasing from 33.87 ± 4.17 to 59.97 ± 13.13. The observed increase in ΔE indicates a marked and perceptible color change, consistent with previous findings that an ΔE value greater than 2.0 is generally distinguishable by the human eye [24]. The ΔE marked a rise, which may be attributed to physiological stress responses, particularly enzymatic browning as well as pigment degradation. A similar trend was observed in the Δa values, which increased from 2.00 ± 0.45 to 3.85 ± 1.05, which reflects a noticeable shift toward the red region of the color spectrum. This trend agrees with earlier findings that associate phenolic accumulation shifts with tuber discoloration. Conversely, the ΔL values showed a substantial decline, decreasing from −27.97 ± 3.19 to −67.25 ± 4.72 in potato tissues. The reduction in ΔL suggests significant darkening, a hallmark of enzymatic browning induced by polyphenol oxidase (PPO) activity, which leads to the polymerization of phenolic compounds such as chlorogenic acid into melanin-like pigment [2]. The decline in Δb, which represents a shift away from 18.95 ± 2.96 to −1.30 ± 0.57, indicates a yellow pigmentation decrease.

The increase in DM content from 17.54 ± 2.48 in the Healthy Group to 23.78 ± 1.46 in the Blackhearted Group reflects notable physiological alterations associated with the development of blackheart disease. The higher DM in the Blackhearted Group likely results from the dehydration and tissue breakdown occurring in the diseased tissues, which leads to a higher concentration of solid matter relative to water content. This increase in DM is consistent with the loss of cellular turgor pressure and the breakdown of water-soluble compounds in the tissues as they deteriorate.

**Figure 4:**
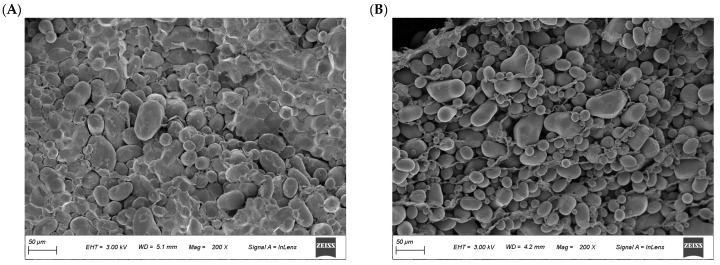
SEM images of the (**A**) Healthy and (**B**) Blackhearted Group.

The SEM micrographs presented in Figure 4 illustrate distinct structural variations between healthy and blackhearted potato tissues. The starch granules appear well defined, densely packed, and uniformly distributed in healthy tissue, reflecting intact cellular organization and strong intercellular cohesion. Blackhearted tissue exhibits irregularly arranged starch granules with low packing density, reduced surface clarity, and partial structural collapse features consistent with tissue degradation driven by enzymatic activity and oxidative stress [2]. This deterioration in structure is likely a consequence of metabolic disruption caused by hypoxic conditions, where reduced respiratory activity compromises membrane integrity and affects the organization of starch granules. Furthermore, the breakdown of pectin and hemicellulose weakens the structural framework of the cell wall.

### 3.2. Comparison of OPs in Two Different Potato Tissues

**Figure 5:**
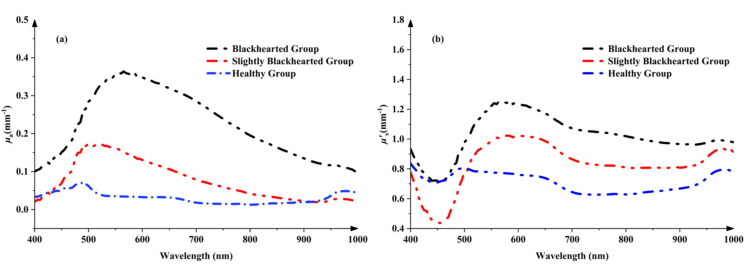
The changes in *μ*_a_ (**a**) and *μ*’_s_ (**b**) in the Healthy Group and Blackhearted Groups.

As shown in Figure 5a, one absorption peak around 490 nm can be observed in the *μ*_a_ spectra of the Healthy Group, which can be attributed to carotenoids [25]. A small peak around 980 nm in the *μ*_a_ spectra corresponded to the combination of water vibrational modes [26]. The peak of *μ*_a_ spectra is around 490 nm in the Healthy Group, possibly due to increased carotenoid content [15]. The changes in *μ*’_s_ values in the Healthy Group and Blackhearted Group are displayed in Figure 5b. It was easily apparent that the average *μ*’_s_ at 400–1000 nm were much higher than their corresponding *μ*_a_ values, primarily because potato tissues possess high scattering properties [27]. The *μ*’_s_ profiles increased with increasing wavelength for 450–550 nm in the blackhearted tissue. In the 550–1000 nm range, the *μ*’_s_ values of blackhearted tissues presented an overall downward trend. The blackhearted tissues exhibited enhanced absorption and scattering, especially in the 500–850 nm range. This provides a basis for the non-destructive optical detection of blackheart disorder at an early stage. Blackheart-affected tissues demonstrated significantly enhanced light absorption and scattering intensity across the 500–850 nm spectral range. This altered optophysical behavior is attributed to two interdependent pathological processes: (1) the progressive disintegration of cellular architecture through enzymatic degradation and (2) the anomalous accumulation of osmotically active compounds increasing the dry matter concentration (Table 2). The enzymatic degradation of cell walls and intracellular constituents in blackhearted tissues results in starch granule accumulation, generating new interfaces for light scattering through structural disorganization. Concurrently, the expansion of intercellular voids (Figure 4) increases the number of air–tissue interfaces, where the pronounced refractive index mismatch further intensifies light scattering. The elevated DM content, potentially caused by starch degradation or moisture loss, increases tissue heterogeneity, enhancing both the absorption and scattering of light. This result is also consistent with the findings of Guo et al. that light absorption by apples increased with increasing apple dry matter content [28].

### 3.3. Discrimination Models for Blackhearted Potato

The comparative analysis of PLS-DA and SVM-DA models for spectral discrimination of blackhearted potatoes (490 and 805 nm) revealed distinct performance patterns contingent on Ops in Table 3. Both models exhibited peak classification accuracy (100.00% overall) in calibration and cross-validation sets when utilizing *T*t and *μ*_a_. Notably, performance diverged significantly across other OPs. While *μ*’_s_-based PLS-DA maintained 80.36% calibration accuracy, its cross-validation reliability declined to 79.17% due to reduced Healthy Group recognition (91.67%). Conversely, *R*t-dependent PLS-DA demonstrated the weakest generalizability, yielding 68.75% cross-validation accuracy. In nearly all scenarios, SVM-DA either matched or outperformed PLS-DA in cross-validation. This highlights the superior robustness of SVM-DA, which exhibited a consistently high and stable cross-validation accuracy range of 95.83% to 100.00% except *R*t.

### 3.4. Light Penetration Depth of Potato Tissues

**Figure 6:**
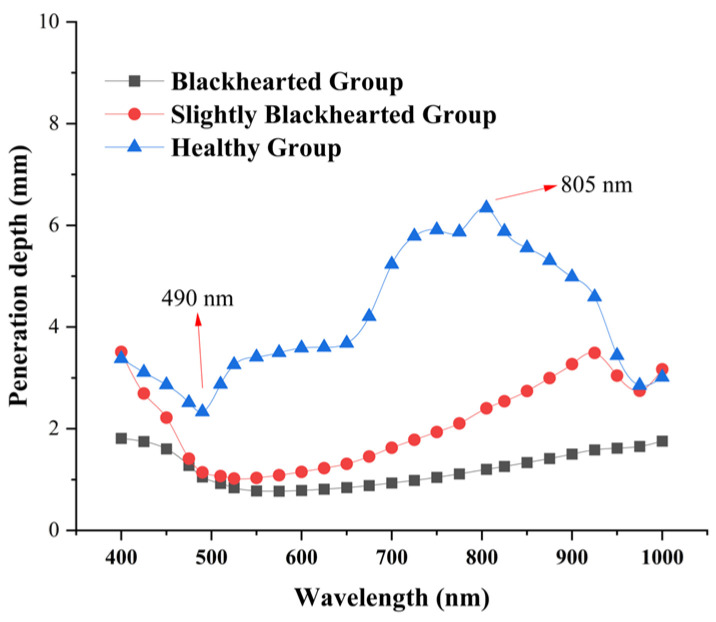
The penetration depth of the Healthy Group, Slightly Blackhearted Group, and Blackhearted Group in potato tissues.

To improve the accuracy and stability of spectral-based non-destructive detection in fruits and vegetables, it is essential to investigate their complex light propagation characteristics, such as scattering, penetration depth, and absorption, to provide a theoretical foundation for internal quality evaluation and system design. As shown in Figure 6, light penetration in both the Healthy and Blackhearted Groups was analyzed across the spectral range of 400–1000 nm. In the Healthy Group, distinct troughs were observed in the penetration depth spectrum, primarily due to the strong absorption of pigments such as carotenoids and water. In contrast, the characteristic carotenoid absorption trough was nearly absent in the Slightly Blackhearted and Blackhearted Groups, consistent with the findings presented in Section 3.2. Notably, the Healthy Group exhibited a relatively high penetration depth between 650 and 950 nm, with values exceeding 3.0 mm. This observation aligns well with the results reported by Zhang et al. [29] and Wang et al. [15].

The most profound penetration depth was at 805 nm in the Healthy Group, approximately 6.73 mm. The wavelengths with the shortest depth penetration in the Healthy Group were 490 nm, approximately 2.17 mm. The most profound penetration depth was 805 nm in the Blackhearted Group, approximately 1.30 mm. In the Healthy group, 490 nm exhibited the shortest penetration depth, reaching approximately 0.45 mm. The difference between the deepest and shortest transmission depths in potatoes in the Healthy Group and Blackhearted Group was 6.04 mm at 805 nm and 1.55 nm at 490 nm, respectively. This may be associated with the chemical changes caused by deterioration, specifically influencing cell structure and increased DM content. For the sake of subsequent comparison, 490 nm and 805 nm were chosen to represent the penetration depth difference.

### 3.5. Validation of Light Propagation Simulations

**Figure 7:**
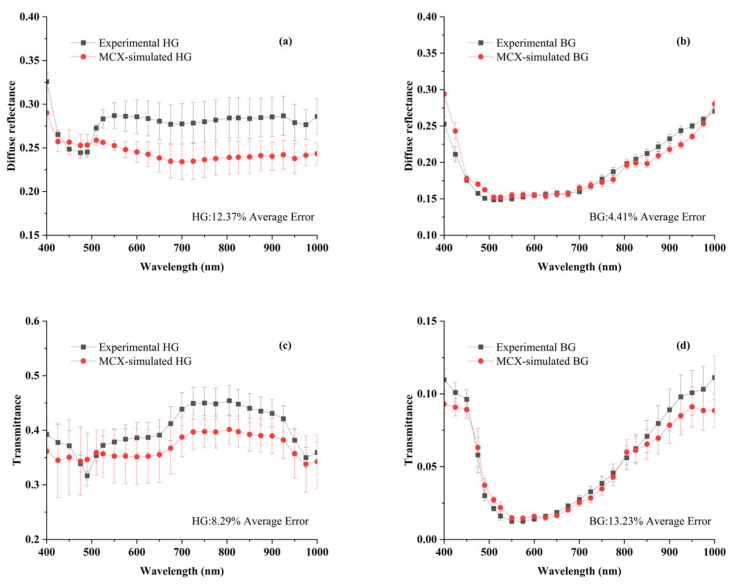
Comparison of the simulated and experimental data for diffuse reflectance and transmittance in the HG (**a**,**c**) and BG (**b**,**d**). The different lines and the error bars represent the average spectrum and the standard deviation of 3 samples. HG: Healthy Group, BG: Blackhearted Group.

The comparison between MCX-simulated and experimental data is presented in Figure 7. Diffuse reflectance and transmittance experimental data were slightly higher or lower than MCX-simulated across the entire wavelength range. The inconsistent results might arise from the IAD algorithm and the statistical nature of the MCX simulation itself [30]. The average simulated reflectance errors for the Healthy and Blackhearted Groups were 12.37% and 4.41%, respectively. The average errors of simulated transmittance for the Healthy Group and Blackhearted Group were 8.29% and 13.23%, respectively. Sample thicknesses and experimental setup might also cause errors in the simulation [31]. Overall, MCX simulations could generate reliable results.

### 3.6. Light Energy Distribution in Potato Tissues

**Figure 8:**
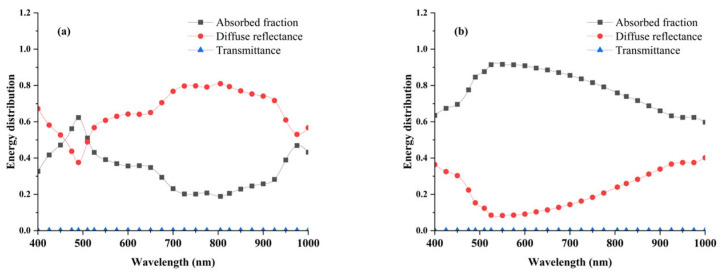
Energy distribution of Healthy Group (**a**) and Blackhearted Group (**b**).

As illustrated in Figure 8a, the energy distribution of the Healthy Group predominantly consists of diffuse reflectance (approximately 65.3%), followed by the absorbed fraction (34.6%), while transmittance remains below 0.1% across the 400–1000 nm spectral range. In contrast, as shown in Figure 8b, the Blackhearted Group exhibits a markedly different energy distribution, with diffuse reflectance reduced to 22.4% and the absorbed fraction increasing to 77.5%, while transmittance remains similarly negligible (<0.1%). A detailed spectral analysis reveals that, in the Healthy Group, the absorbed fraction undergoes a noticeable increase at specific wavelengths, particularly around 490 nm and 975 nm. Furthermore, a higher absorption fraction is consistently observed in the Blackhearted Group across the entire 400–1000 nm range, with a particularly pronounced increase in the 500–800 nm region, where the absorption fraction is significantly greater than that of the Healthy Group. The highest absorption fraction for the Healthy Group is recorded in the 720–900 nm region, aligning with expected spectral behavior. Moreover, the spectral trend of the absorption fraction closely follows the spectral pattern of the *μ*_a_, as presented in Figure 5, further reinforcing the correlation between optical properties and energy distribution in potato tissues. The transmittance in the energy distribution is less than 0.1% (400–1000 nm) when the thickness of the sample is 2 mm, demonstrating that only a tiny fraction of light could pass through the potato tissue. Most of the incident energy was absorbed or reflected within the tissue; this limitation can be mitigated by increasing the input light intensity [27]. Most of the energy was absorbed or reflected in potato tissues, which is consistent with Wang et al. [12,21].

### 3.7. Absorption Energy Density Distribution in Flavedo Tissues

**Figure 9:**
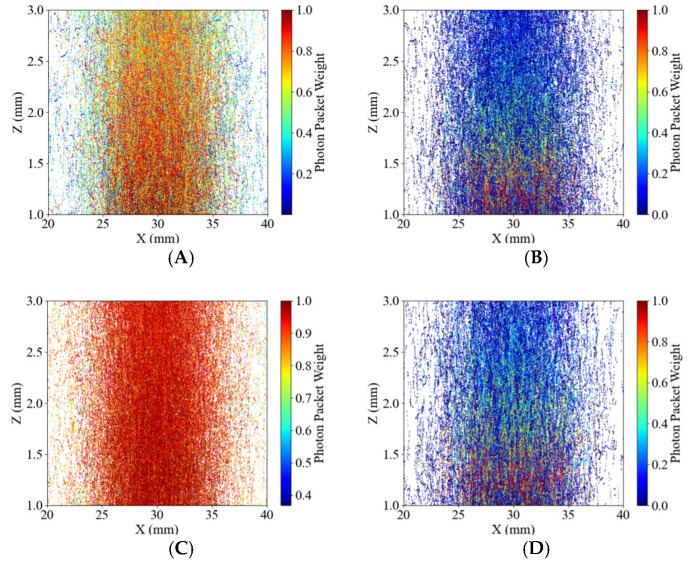
Profile of photon packets propagating in the 2 mm potato tissues. The color bar indicates the weight of the photon packets. (**A**) 490 nm for the Healthy Group, (**B**) 490 nm for the Blackhearted Group, (**C**) 805 nm for the Healthy Group, and (**D**) 805 nm for the Blackhearted Group.

The 490 and 805 nm wavelengths were selected and compared with the propagation of photon packets at the corresponding wavelengths (Figure 9A–D). Compared with the energy distribution at 490 nm of healthy potatoes, it could be found that the photon packet weight more significantly decreased at 490 nm in blackhearted tissues than in healthy tissues, corresponding to the lower transmittance depth around 490 nm in Figure 6. This indicates that the blackhearted tissues have a compromised ability to transmit light. In contrast, at 805 nm (Figure 9C,D), the photon packet weight remained consistently high across a broader spatial region in healthy tissue, suggesting enhanced photon propagation at longer wavelengths. However, a notable reduction in the density and weight of photon packets was still observed in blackhearted tissue at the same wavelength. Despite the longer wavelength’s deeper tissue penetration capability, light attenuation remained evident in diseased regions. These results demonstrate wavelength-dependent differences in photon transport behavior between healthy and blackhearted potato tissues, consistent with Yang and Guo et al. [12,21,24]. While longer wavelengths (e.g., 805 nm) exhibit improved penetration and more uniform photon distribution, tissue degeneration in the blackhearted samples still substantially impairs light transmission. This wavelength-selective attenuation provides a theoretical basis for spectral region optimization in non-destructive optical diagnostics of internal potato defects.

**Figure 10:**
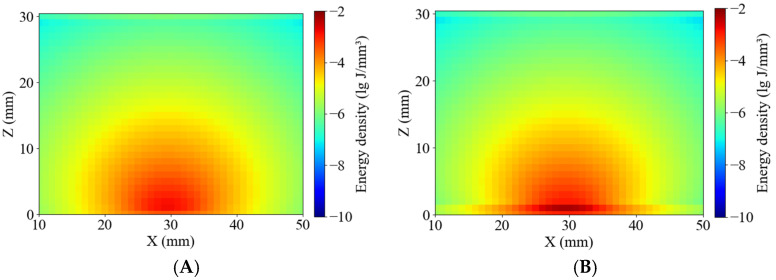

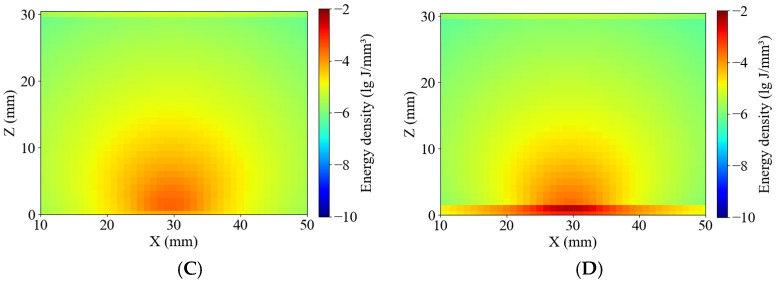
Energy distribution of the Healthy Group and Blackhearted Group of halved potatoes. (**A**) 490 nm for the Healthy Group, (**B**) 490 nm for the Blackhearted Group, (**C**) 805 nm for the Healthy Group, and (**D**) 805 nm for the Blackhearted Group.

As shown in Figure 10, the simulated absorption energy density distributions were compared between healthy and blackhearted potato tissues at 490 nm and 805 nm. The energy distribution patterns were consistent with those reported by Yang et al. and Guo et al. [23,24], exhibiting relatively homogeneous margin lines in both tissue types. Figure 6 and Figure 10 show that greater penetration depth was associated with lower tissue absorption [31]. At 490 nm, where the penetration depth was lower, photon packet energy rapidly attenuated near 15 mm, resulting in shallow energy contours, especially in the blackhearted tissue. The margin lines extended significantly, and were not clear at 805 nm, due to reduced absorption in healthy tissues, whereas 490 nm and 805 nm showed broader energy distributions than blackhearted tissues, indicating a lower absorbance fraction and greater optical transparency.

**Figure 11:**
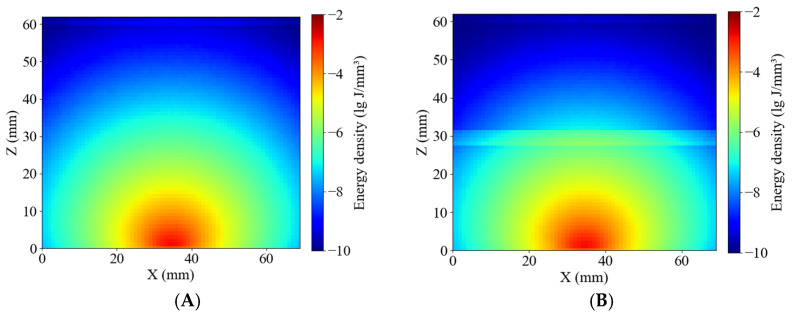

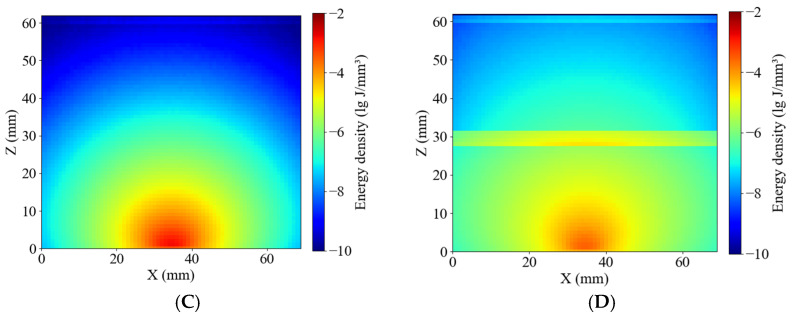
Energy distribution of the Healthy Group and Blackhearted Group of whole potatoes. (**A**) 490 nm for the Healthy Group; (**B**) 490 nm for the Blackhearted Group; (**C**) 805 nm for the Healthy Group; (**D**) 805 nm for the Blackhearted Group.

Figure 11 presents the simulated energy density distributions within whole-potato models for the Healthy and Blackhearted Groups under 490 nm and 805 nm. These results reflect how internal tissue composition and optical properties influence photon propagation and absorption on a volumetric scale. The energy density in healthy tissue (Figure 11A) is primarily concentrated near the incident region and gradually attenuates with increasing depth at 490 nm, forming concentric contours. In contrast, the blackhearted tissue (Figure 11B) shows an abrupt reduction in energy at approximately 30 mm depth, which demonstrates the location of the blackhearted core—the strong absorption caused by structural degradation results in significant photon energy loss. The light penetrates more deeply in both groups due to the lower absorption coefficient at 805 nm. In Figure 11C, the energy distribution of healthy tissue extends further, indicating efficient photon propagation and low attenuation. However, in the Blackhearted Group (Figure 11D), an apparent energy discontinuity is again observed at the depth of the blackhearted region, though less pronounced than at 490 nm. The blackhearted tissue can hinder photon propagation at 409 nm and 805 nm. The Healthy Group exhibited smoother and more symmetric energy distribution patterns at 409 nm and 805 nm, while the Blackhearted Group showed distinct regions of localized energy attenuation. These anomalies suggest that tissue necrosis and structural deterioration enhance photon absorption.

## 4. Discussion

This study demonstrates that the OPs of blackhearted potato tissues exhibit distinct changes compared with healthy tissues, particularly in *μ*_a_. The increase in *μ*_a_ values within the 500–850 nm range indicates heightened absorption, aligning with previous studies on diseased plant tissues. Unlike prior findings suggesting a decrease in *μ*’_s_ due to tissue softening, our results showed an increase in *μ*’_s_ in BH potatoes. This suggests that blackheart disorder leads to microstructural changes, including higher dry matter content, which enhances light scattering.

MC simulations revealed a significantly lower light penetration depth in BH tissues than in healthy ones, with the deepest penetration at 805 nm. The differences between simulated and experimental results may be due to algorithmic approximations and sample thickness variations [15]. These results confirm that physiological changes caused by blackheart disorder directly affect light transport properties in potato tissues.

PLS-DA and SVM-DA models demonstrated exceptional accuracy in distinguishing blackhearted from healthy potatoes, underscoring the robustness of spectral features in defect detection. This aligns with previous research on internal defect classification using Vis-NIR spectroscopy. The application of *T*t, *R*t, *μ*_a_, and *μ*’_s_ in modeling improves performance, suggesting that *T*t and *μ*_a_ are optimal for early detection.

The strong absorption observed at 490 nm is likely related to pigment breakdown, whereas the deeper tissue penetration at 805 nm offers meaningful insights into internal structural changes, identifying 490 nm and 805 nm as critical wavelengths for detecting blackheart in potatoes. These findings support the development of improved nondestructive quality assessment techniques, emphasizing reflection-based methods for practical applications in the potato industry.

Despite promising results, this study has limitations. The MC simulations were based on homogeneous tissue models, which may not fully capture complex potato structures. Future research should explore multilayered MC models for improved accuracy. Extending the study to more potato varieties and integrating 3D imaging could enhance detection capabilities. These findings lay the groundwork for advancing agricultural quality control and reducing postharvest losses.

Furthermore, the results indicate that Vis-NIR spectroscopy has the potential to enable non-destructive detection of blackheart disorder through the intact skin of potato tubers. The observed spectral penetration, particularly around 805 nm, suggests photons can reach internal defective regions without needing physical slicing. This provides a promising theoretical foundation for developing through-skin optical sensing systems, which could significantly improve industrial grading and early defect screening of potatoes.

## 5. Conclusions

This study systematically examines the optical properties and MC simulations of light propagation in healthy and blackhearted potatoes, shedding light on the distinct differences in optical behavior between the two groups. Detailed analysis revealed that blackhearted tissues, characterized by their physiological degradation, exhibit markedly lower photon packet weight and reduced penetration depth. Specifically, the light penetration in blackhearted tissues was significantly shallower than that in healthy tissues, indicating a notable alteration in diseased tissues’ internal light transport properties. This was further corroborated by MC simulations, which demonstrated that the photon penetration depth in healthy tissues reached up to 6.73 mm at a wavelength of 805 nm, while in blackhearted tissues, it was limited to a mere 1.30 mm at the same wavelength.

Additionally, the study revealed that the absorption energy at both 490 nm and 805 nm wavelengths was significantly higher in blackhearted tissues. This suggests that these wavelengths may be particularly effective for detecting the presence of blackheart, offering the potential for improved non-invasive detection techniques. The findings from Vis-NIR spectroscopy also aligned with the simulation results, with blackhearted tissues exhibiting enhanced absorption and reduced scattering, especially in the 500–850 nm range. These changes in optical properties are crucial for understanding the underlying physiological alterations in blackhearted potatoes. Furthermore, the study utilized advanced classification models, specifically PLS-DA and SVM-DA, to discriminate between healthy and blackhearted tissues based on *T*t, *R*t, *μ*_a_ and *μ*’_s_. The SVM-DA models demonstrated exceptional performance, achieving 95.83-100.00% accuracy in Cross-Validation sets, thereby confirming the robustness and reliability of the optical features for accurate blackheart detection.

This study advances the understanding of light transport dynamics in potato tissues by identifying key optical distinctions between healthy and blackhearted samples. The findings establish a robust theoretical framework for enhancing the precision and applicability of non-destructive detection techniques, with potential implications for improving agricultural quality control and early defect identification. Furthermore, the demonstrated spectral penetration, particularly near 805 nm, supports the feasibility of through-peel detection of blackheart disorder, laying a solid foundation for the development of future real-time inspection systems.

## Figures and Tables

**Table 1:** Input parameters for Monte Carlo simulation of light propagation in potatoes.

Parameters	Values
Number of photons	100,000,000
Voxel size	0.5
Refractive index for medium above and below (air)	1.0
Refractive index of potato	1.34
Number of voxels for both x and y	60
Number of voxels for depth z	2/30/60

**Table 2:** The changes in physiological indicators of potatoes.

Group	ΔL	Δa	Δb	ΔE	DM (g/100 g)
Healthy	−27.97 ± 3.19	2 ± 0.45	18.95 ± 2.96	33.87 ± 4.17	17.54 ± 2.48
Blackhearted	−67.25 ± 4.72	3.85 ± 1.05	−1.3 ± 1.57	59.97 ± 13.13	23.78 ± 1.46

**Table 3:** The comparative analysis of PLS-DA and SVM-DA models of healthy and blackhearted potatoes.

Models	Optical Parameters	Calibration Set (%)			Cross-Validation Set (%)		
		Healthy	Blackhearted	Overall	Healthy	Blackhearted	Overall
PLS-DA	*T*_t_	100.00	100.00	100.00	100.00	100.00	100.00
	*R*_t_	51.79	82.29	70.54	45.83	91.67	68.75
	*μ*_a_	100.00	100.00	100.00	100.00	100.00	100.00
	*μ*’_s_	60.71	100	80.36	58.33	100	79.17
SVM-DA	*T*_t_	100.00	100.00	100.00	100.00	100.00	100.00
	*R*_t_	51.79	94.64	73.21	45.83	100.00	72.92
	*μ*_a_	100.00	100.00	100.00	100.00	100.00	100.00
	*μ*’_s_	100	100	100	91.67	100	95.83

## Data Availability

Data will be made available upon request.
